# Semiparametric Accelerated Failure Time Model as a New Approach for Health Science Studies

**Published:** 2017-11

**Authors:** Mostafa KARIMI, Ardalan SHARIAT

**Affiliations:** 1. Institute for Mathematical Research, University Putra Malaysia, Serdang, Selangor, Malaysia; 2. Sports Medicine Research Center, Neuroscience Institute, Tehran University of Medical Sciences, Tehran, Iran

## Dear Editor-in-Chief

Time to event data, also known as survival time data or failure time data are very common in medical studies and health science research. The event of interest in such data may be positive, such as recovery or restoration; negative, such as heart attack or death; or neutral, such as renew or changing medical prescriptions. The distinguishing feature of time to event data is that for some patients the survival time or failure time may be censored. Censored observation arises if at the end of the follow-up period a patient does not experience the event of interest. In such cases, it is unknown that whether the patients experience the event after the end of the observation time.

Making inference for time to event data cannot be carried out through commonly used linear models, such as linear regression or parametric analysis of variance (1). Precisely, since the data includes censored observations, the necessary assumptions of linear models like linearity or normality cannot be met. For analyzing time to event data in the presence of censored observations the accelerated failure time (AFT) model is a very useful model which relates a set of predictors or independent variables to the logarithm of survival or failure time (2). In particular, AFT model indicates that for *n* patients,

$$\text{log}\;T_{i}=\beta_{1}X_{i1}+\beta_{2}X_{i2}+\cdots +\beta_{k}X_{ik}+\varepsilon_{i}(i=1,2,\ldots ,n)$$

where *T*_*i*_ is the survival time or failure time of the *i*th patient, *β*_1_, *β*_2_, ..., *β*_*k*_ are unknown regression parameters of the model, and *ε*_*i*_’s are error terms.

An AFT model assumes that the effect of a risk factor or an independent variable is to accelerate or decelerate the survival time or failure time by some constant.

If the probability distribution of the error terms in the model is one of the well-known statistical distributions then the AFT model is called parametric, otherwise, the AFT model is semi-parametric. The commonly used distributions for parametric AFT model are log-logistic, exponential, and Weibull. Regression parameters in a parametric AFT model can be estimated through maximum likelihood estimation, while the parameter estimates in a semiparametric AFT model can be obtained using rank-based estimators (3).

Although the maximum likelihood estimation in parametric AFT models is theoretically simple, checking the assumptions about the statistical distribution of the error terms and goodness of fit tests for survival times or failure times are usually problematic and sometimes theoretically complicated (4). The main advantage of semi-parametric AFT models in health science studies is that such inferences do not involve any assumptions about the probability distribution of the error terms in the model. Moreover, in semi-parametric AFT models the empirical distribution of the parameter estimators can be easily estimated, so constructing the confidence intervals for the regression parameters or hypothesis testing can be carried out through simple and reliable computations (5).

As a home message for the researchers in the field of health sciences, when time to event data in a medical study include censored observations linear regression models are not useful, so an AFT model could be used for modeling the effect of independent variables on the survival time or failure time as dependent variable. Since the parametric AFT models make assumptions about the probability distribution of the model error terms that are not easy to satisfy, the inference about the model and estimating the regression parameters could be carried out through a semiparametric AFT model. The rank-based inference procedures for semiparametric AFT models are widely studied and highly recommended for estimating the model parameters due to their simple and reliable computational methods in applications.
